# Protease-activated receptor 2 promotes clearance of *Pseudomonas aeruginosa* infection by inducing cAMP-Rac1 signaling in alveolar macrophages

**DOI:** 10.3389/fphar.2022.874197

**Published:** 2022-09-20

**Authors:** Sheikh Rayees, Jagdish Chandra Joshi, Bhagwati Joshi, Vigneshwaran Vellingiri, Somenath Banerjee, Dolly Mehta

**Affiliations:** Department of Pharmacology and Regenerative Medicine, Centre for Lung and Vascular Biology, College of Medicine, University of Illinois, Chicago, IL, United States

**Keywords:** acute lung injury, cAMP, *Pseudomonas aeruginosa*, alveolar macrophage, PAR2, Rac1

## Abstract

Efficient phagocytosis of pathogens by the innate immune system during infectious injury is vital for restoring tissue integrity. Impaired phagocytosis, such as in the case of infection with *Pseudomonas aeruginosa*, a broad-spectrum antibiotic-resistant Gram-negative bacterium, can lead to a life threatening lung disorder, acute lung injury (ALI). Evidence indicates that loss of protease-activated receptor 2 (PAR2) impaired *Pseudomonas aeruginosa* clearance leading to non-resolvable ALI, but the mechanism remains unclear. Here, we focused on the alveolar macrophages (AMs), the predominant population of lung-resident macrophages involved in sensing bacteria, to understand their role in PAR2-mediated phagocytosis of *Pseudomonas aeruginosa*. We found that upon binding *Pseudomonas aeruginosa*, PAR2-expressing but not PAR2-null AMs had increased cAMP levels, which activated Rac1 through protein kinase A. Activated Rac1 increased actin-rich protrusions to augment the phagocytosis of *Pseudomonas aeruginosa*. Administration of liposomes containing constitutively active Rac1 into PAR2-null mice lungs rescued phagocytosis and enhanced the survival of PAR2-null mice from pneumonia. These studies showed that PAR2 drives the cAMP-Rac1 signaling cascade that activates *Pseudomonas aeruginosa* phagocytosis in AMs, thereby preventing death from bacterial pneumonia.

## Introduction

Macrophages are the professional phagocytes of the innate immune system involved in host defense (Freeman and Grinstein, 2016). Phagocytosis of apoptotic cells and pathogens by the macrophages is essential for restoring the integrity of infectious tissue (Freeman and Grinstein, 2016; Han et al., 2016). Phagocytosis is a multistep process that involves the sensing of pathogens followed by their binding to the phagocytic cells for subsequent internalization (Levin et al., 2016).

The tissue-resident alveolar macrophages (AMs) are unique because they directly communicate with the environment and defend the lungs against broad-spectrum and antibiotic-resistant species of Gram-negative bacteria, including *Pseudomonas aeruginosa* (*PA*, hence forth) (Restrepo et al., 1999; Moraes et al., 2008; Nagre et al., 2019). *PA*, being an extracellular pathogen, prefers to associate with the surface of the host cell membrane (Lillehoj et al., 2001; Siryaporn et al., 2014). Mostly, opsonic receptors phagocytized *PA*, but AMs express these receptors at relatively low levels (Stokes et al., 1998; Lovewell et al., 2014). Hence, *PA* infections can lead to acute lung injury, a life-threatening respiratory disorder characterized by severe alveolar–capillary barrier leakage and inflammation. Also, *PA* can cause lethal infections in cystic fibrosis patients (Malhotra et al., 2019). Therefore, understanding mechanisms promoting *PA* clearance by AMs will potentially identify targets for suppressing diseases post infection.

In the present study, we focused on the protease-activated receptor 2 (PAR2) expressed on AMs as a possible mechanism inducing phagocytosis of *PA*. PAR2 is a G protein-coupled receptor belonging to the protease-activated receptor (PAR) family (Rayees et al., 2019). Proteases released during injury, such as thrombin, activate PAR2 (Zhao et al., 2015; Rayees et al., 2019). Interestingly, the proteases secreted by *PA* inactivate PAR2 (Dulon et al., 2005; Moraes et al., 2008). Mice lacking PAR2 showed severe lung inflammation and reduced *PA* clearance (Moraes et al., 2008). These authors also showed that *PA* infection increased neutrophil transmigration in PAR2-null lungs (Moraes et al., 2008). Because neutrophils require AM functions to clear *PA* (Mishra et al., 2012; Thanabalasuriar et al., 2017), it is unclear how the loss of PAR2 in AMs signals impaired phagocytosis of *PA*.

Here, we showed that upon binding *PA*, PAR2^+^ AMs increases cAMP, which in turn activates the small GTPase Rac1. Activated Rac1 forms actin-rich protrusions to phagocytize *PA*. Thus, AMs lacking PAR2 could not efficiently clear *PA*, and as a result, PAR2-null mice showed significant mortality after pneumonia. Liposomal delivery of constitutively active Rac1 into PAR2-null mice rescued phagocytosis and prevented their mortality. The studies raise the possibility of targeting the PAR2-Rac1 cascade as a tool to promote *PA* phagocytosis, thereby increasing bacterial clearance and hastening the resolution of pneumonia.

## Material and methods

### Experimental animals

All animal experiments were approved by the Institutional Animal Care and Use Committee of the University of Illinois at Chicago. C57BL/6J (WT, henceforth) and PAR2-null mice breeding pairs (Jackson Laboratory, CT, United States) were bred at the University of Illinois at Chicago. The mice colonies were maintained in a pathogen-free housing facility at the University of Illinois. For the experiments, 6–8-week-old male and female mice were used. Data represent pooled data from both female and male mice as we did not observe any effect of gender on lung injury. The wild-type strain of *Pseudomonas aeruginosa* (PAO1) constitutively expressing GFP (GFP-PAO1) was used in the study. The expression of GFP was plasmid-based as described previously (Hashimoto et al., 2007). The pharmacological agents NSC23766, Y-27632, and 8-bromoadenosine 3′-, 5′-cyclic monophosphate (8-Br-cAMP) were dissolved in water, whereas H-89 and ESI-09 were dissolved in DMSO.

### *Pseudomonas aeruginosa* culture and count

GFP-tagged *PA* (GFP-PAO1 strain) was streaked in ampicillin-selective (HiMedia Laboratories LLC, United States) Luria–Bertani (LB) agar plates and incubated overnight at 37°C from the glycerol-preserved vial for the activation of culture. Single colonies were picked and inoculated overnight (∼17 h) in 250 ml LB broth (Amp 100 μg/ml) at 37°C. For the standard plate count method, 1 ml of the bacterial culture from the broth was serially diluted with sterile phosphate buffer up to 1 × 10^10^ dilutions. Each dilution plate was spread-plated in ampicillin-selective (100 μg/ml) sheep blood agar plates to get the countable bacterial colonies. The 1 × 10^6^ CFU/25uL was calculated using the following formula: CFU/ml = (no. of colonies x dilution factor)/volume of the culture plate.

### *Pseudomonas aeruginosa*-induced acute lung injury

Acute lung injury was induced in the WT and PAR2-null mice post *PA* infection and determined as described previously (Joshi et al., 2020). The mice were anesthetized by intraperitoneal injections of ketamine (100 mg/kg) and xylazine (12 mg/kg). *PA* (1 × 10^6^ CFU in 50 ul PBS/mouse) was instilled into the lungs through the endotracheal route. Edema was measured in these mice at indicated times by measuring the lung wet–dry ratio. Briefly, the left lobe of the lungs was excised following *PA* instillation, weighed, and then dried at 55°C. The dry weight was taken after 24 h, and the wet–dry ratio was calculated, which gave a measure of edema, as described previously (Yazbeck et al., 2017).

### Bronchoalveolar lavage

Bronchoalveolar lavage **(**BAL) and BAL-AMs were isolated as described previously (Rayees et al., 2019; Joshi et al., 2020). Briefly, after sacrificing the mice, tracheotomy was performed followed by the collection of BAL using an 18-gauge blunt needle. The BAL fluid was poured on plastic dishes, supplied with 10% serum, and the AMs allowed to adhere for 60 min. The non-adherent cells were removed by washing with PBS.

### Phagocytosis assays

Phagocytosis assays in bone marrow-derived macrophages (BMDMs) or AMs were performed as described previously, with modifications (Ojielo et al., 2003; Descamps et al., 2012; Bastaert et al., 2018; Nagre et al., 2019). BMDMs were isolated as described previously (Rayees et al., 2019) and exposed to *PA* at MOI (multiplicity of infection) 1:10 in an antibiotic-free media containing 1% FBS for indicated times. The experiment was terminated by washing out *PA* with PBS. For FACS analysis, for the measurement of gene expression or immunoblotting, the cells were detached from the surface using accutase and processed further as described. For immunofluorescence, the macrophages were fixed in 2% paraformaldehyde for 15 min and stained with phalloidin (1:100) for 1 h and imaged. For AM phagocytosis, BAL-AMs were exposed to *PA* (MOI, 1:10) for 60 min. The AMs were fixed in 2% paraformaldehyde for 15 min after which cells were permeabilized using 0.1% triton X and stained with phalloidin (1:100) for 1 h in dark. The AMs and BMDMs were imaged on an LSM880 confocal microscope using 63x oil objective. F-actin organization in the form of pseudopods was quantified as the ratio of pseudopods/actin projections toward *PA*.

For measuring Rac1 activity in AMs by immunofluorescence, we quantified pseudopods, well-known indices of Rac1 activity (Sit and Manser, 2011; Flannagan et al., 2012), by measuring the spatial distribution of Rac1/F-actin fluorescent intensity peaks near cell edges. Briefly, the individual cells with protruding edges expressing fluorescent-labeled Rac1/F-actin were segmented, and the fluorescence intensity was measured using *ImageJ*.

### Bacterial internalization assay

WT and PAR2-null BMDMs were exposed to *PA* for 45 min. The supernatant was centrifuged and dissolved in 50 μL sterile PBS. The BMDM monolayer was washed twice with PBS and incubated with gentamicin-containing (300 μg/ml) media for 1 h to kill non-internalized bacteria (Plotkowski et al., 1999). Following a PBS wash, the cell pellet was then lysed in 0.1% Triton X-100 (Sigma-Aldrich, United States). The dissolved supernatant and the cell lysate were spread on the blood agar plate, and *PA*-CFU was counted 24 h later (Almeida et al., 1999; Cavinato et al., 2020).

### FACS analysis

The BMDMs exposed to *PA* for indicated time points were washed twice with PBS. The cells were detached from the plates using accutase, centrifuged, and fixed with fixation buffer (1:1 with PBS) (Invitrogen, United States) for 20 min. The cell suspension was washed twice with FACS buffer (0.5% BSA in PBS). All samples were then run on the BD LSR-Fortessa flow cytometer (BD Biosciences, CA, United States). The data were processed using Flow Jo software (TreeStar, Inc., CA, United States).

### Rac1 activity and immunoblotting

The BMDMs were exposed to *PA* at MOI, 1: 10 for indicated times. Cells were lysed using RIPA buffer (Sigma-Aldrich, United States) containing 1% protease inhibitor cocktail. Lysates (120 μg protein) were incubated with PAK-GST protein beads (20 μg) (Cytoskeleton, Inc., United States) for 4 h at 4°C on gentle rotation. The samples were centrifuged, and the pellet containing the beads was washed carefully and dissolved in Laemmli buffer. The total and active Rac1 were assessed by immunoblotting, using the anti-Rac1 monoclonal antibody.

### Transfection

The BMDMs were transfected as described previously (Rayees et al., 2019), using the Amaxa Nucleofactor electroporation system (Lonza). Briefly, the cells were detached from the dishes using accutase and spun down. The cell pellet was suspended in transfection reagent containing cDNA. The cells were slowly mixed with the transfection reagent to apply cDNA to the cells and electroporated. Vector plasmids were used to transfect experimental controls. The electroporated cells were processed for further experiments 48 h after transfection.

### Measurement of gene expression

The RNA was extracted from AMs and BMDMs using TRIzol (Invitrogen Inc., CA, United States). The quality and integrity of RNA were verified on the NanoDrop spectrometer (Thermo Fisher Scientific, MA, United States). The cDNA was synthesized as per manufacturer’s protocol (High-Capacity cDNA Reverse Transcription Kit, Applied Biosystems), using equal quantities of RNA. Real-time q-PCR was performed for the measurement of gene expression (Applied Biosystems, CA, United States) (Rayees et al., 2015). The primer sequences used in the study were taken from our previously published study (Rayees et al., 2019).

### Liposome mediated cDNA delivery to the mouse lungs

Cationic liposomes were made using a mixture of chloroform, cholesterol, and dimethyl dioctadecyl ammonium bromide, as described previously (Yazbeck et al., 2017; Rayees et al., 2019). The liposomes were filtered through a 0.45-micron filter, and the cDNA was gently added and mixed. The cDNA-loaded liposomes were injected into the mouse lungs through the endotracheal route, followed by *PA* administration after 24 h. The lungs were either collected for the measurement of the wet–dry ratio or BAL was performed for the bacterial colony count, immunofluorescence, or measurement of gene expression from the extracted AMs.

### Statistical analysis

All values are given as mean ± SD. Statistical analysis was performed using Graph Pad Prism version 7.0 (Graph Pad Software, La Jolla, CA). Multiple groups were compared using One-way ANOVA followed by a paired Student’s t test to assess significance between two groups. The statistics details can be found in the individual figure legends.

## Results

### Macrophage PAR2 enables phagocytosis of *PA*

**FIGURE 1:**
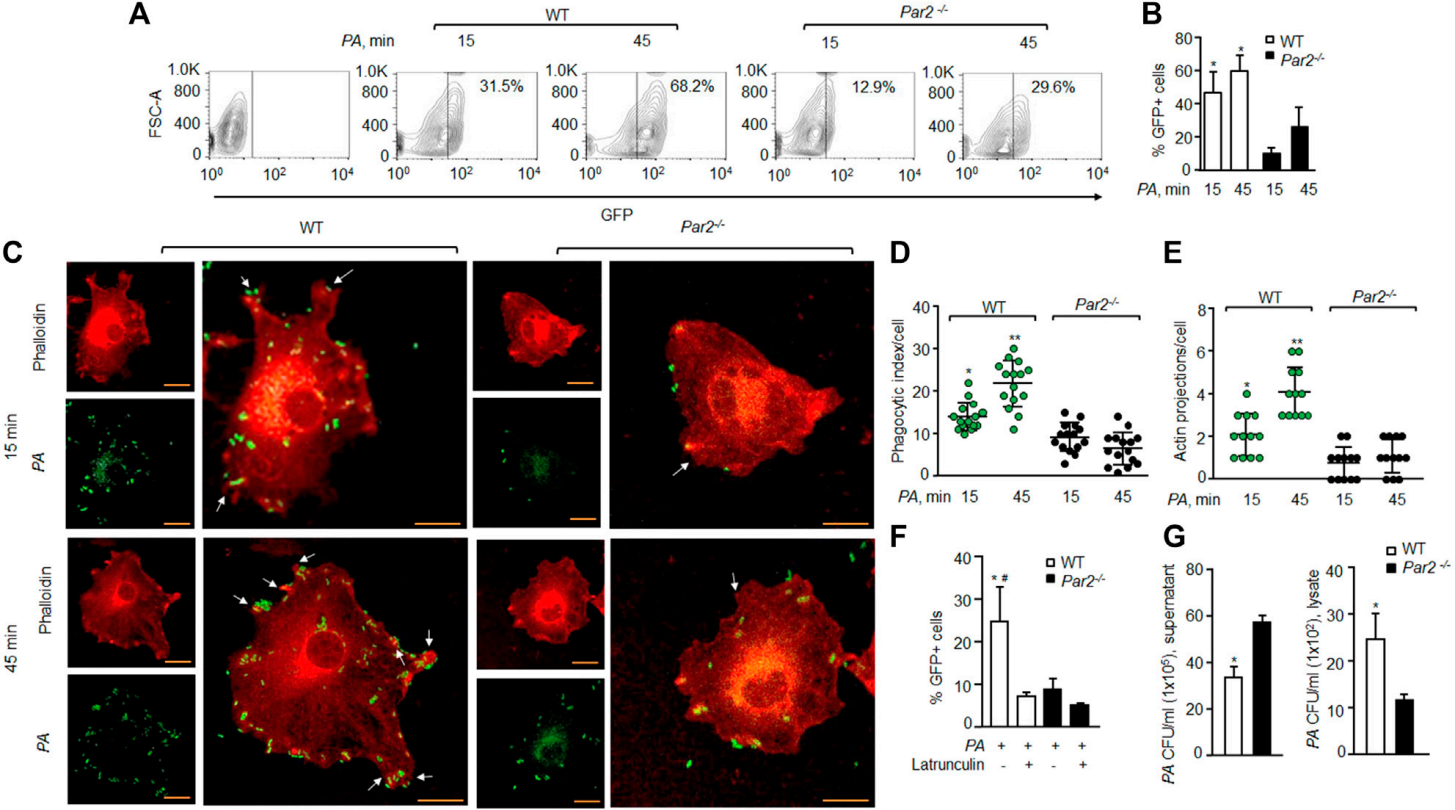
Loss of PAR2 impairs *PA* phagocytosis. **(A)** Macrophages were exposed to GFP-labeled *PA* (MOI, 1:10) for indicated times and then treated with accutase, spun down, and washed. The GFP^+^ cells were gated to assess phagocytosis. A representative contour plot is shown from experiments that were performed three times. **(B)** Mean ± SD of GFP^+^ macrophages, following *PA* exposure, is shown (from the aforementioned experiment-A) at indicated times from three independently performed experiments. **(C)** Macrophages were exposed to *PA* (MOI, 1:10) for indicated times and fixed with 2% paraformaldehyde. The cells were stained with rhodamine-phalloidin and imaged. Representative images are shown from the experiments that were performed three times. Bars, 5 micron meter, inset, zoomed ∼x2. **(D)** Phagocytic index per macrophage (of Figure 1C) was calculated by counting the total number of bacteria adhered to/engulfed by a macrophage. Individual count/phagocytic index per macrophage is shown (n = 15 cells). **(E)** Protrusions (of Figure 1C) were quantified per macrophage. The data are shown as individual extensions per macrophage (n = 12 cells). **(F)** WT and PAR2-nullpaired macrophages were pretreated with latrunculin (20 nΜ for 30 min) after which they were exposed to *PA* for 45 min. The GFP^+^ cells were gated and quantified by flow cytometric analysis. Data are shown as mean of three independent experiments. **(G)** WT and PAR2-null macrophages were exposed to *PA* for 45 min, and the supernatant and cell lysates were collected, dissolved in sterile PBS and spread on blood agar plates. CFU of *PA*/ml were determined. Data are shown as mean of three independent experiments. In all figures, data are presented as mean ± SD. *p < 0.05, **p < 0.001 relative to respective PAR2-null *PA-*exposed macrophages; #*p* < 0.05 relative to latrunculin-treated WT macrophages. The analysis was performed using one-way ANOVA followed by a paired *t*-test.

We exposed bone-marrow-derived Mɸ (macrophage) isolated from the WT and PAR2-null mice to the GFP-tagged *PA* (MOI 1:10) to determine the role of PAR2 in regulating phagocytosis of *PA* by flow cytometry. We found that the mean phagocytic index of WT-Mɸ was 47.2% at 15 min, which increased further to 60.4% at 45 min. In contrast, the mean phagocytic index of PAR2-deficient macrophages was only 11% and 26.5% at 15 and 45 min, respectively (Figures 1A,B). Because phagocytosis requires actin remodeling (Flannagan et al., 2012; Levin et al., 2016), we labeled F-actin using rhodamine-phalloidin to determine the effect of PAR2 deletion on the *PA*-induced actin reorganization. We observed that the phagocytic index of WT macrophages was ∼55% higher than that in PAR2 null-Mɸ (Figures 1C,D). The formation of membrane protrusions is an important aspect of phagocytosis, which is generated by the actin cytoskeleton in response to a phagocytic target (Flannagan et al., 2012; Levin et al., 2016). We quantified the protrusions as the ratio of pseudopods/actin projections toward *PA*. We found that WT-Mɸ extended pseudopods (two to four per cell) around *PA* within 15–45 min (Figure 1E). However, these pseudopods were barely detectable in PAR2-null Mɸ (Figure 1E), recapitulating the aforementioned findings. We next pre-treated the WT or PAR2 null Mɸ with latrunculin A, which is known to block actin polymerization (Morton et al., 2000), followed by *PA* exposure. We found that latrunculin A reduced phagocytosis of *PA* in WT-Mɸ to the level seen in PAR2-null Mɸ (Figure 1F, Supplementary Figure S1A). These findings indicate that PAR2-mediated phagocytosis of *PA* required actin polymerization.

Phagocytosis is a multi-step process initiated upon sensing pathogens, followed by phagosome formation (Flannagan et al., 2012; Levin et al., 2016). We, therefore, determined the number of colony-forming units (CFUs) in the supernatant or lysates of the WT or PAR2-null Mɸ following *PA* exposure to evaluate whether the impaired phagocytosis seen later was due to a defect in bacterial internalization by the Mɸ. CFUs were quantified in lysates following gentamycin treatment to remove attached bacteria. We found that the supernatant from PAR2-null Mɸ formed significantly more *PA* colonies than that from WT-Mɸ (Figure 1G, left), indicating a defective internalization of *PA* by PAR2-null macrophages as a significant factor in reducing phagocytosis. This conclusion is supported by Figures 1C–E data also. Conversely, PAR2-null Mɸ lysates formed fewer *PA* colonies than WT-Mɸ (Figure 1G, right).

### Loss of PAR2 induces bacterial pneumonia and lethality in mice

**FIGURE 2:**
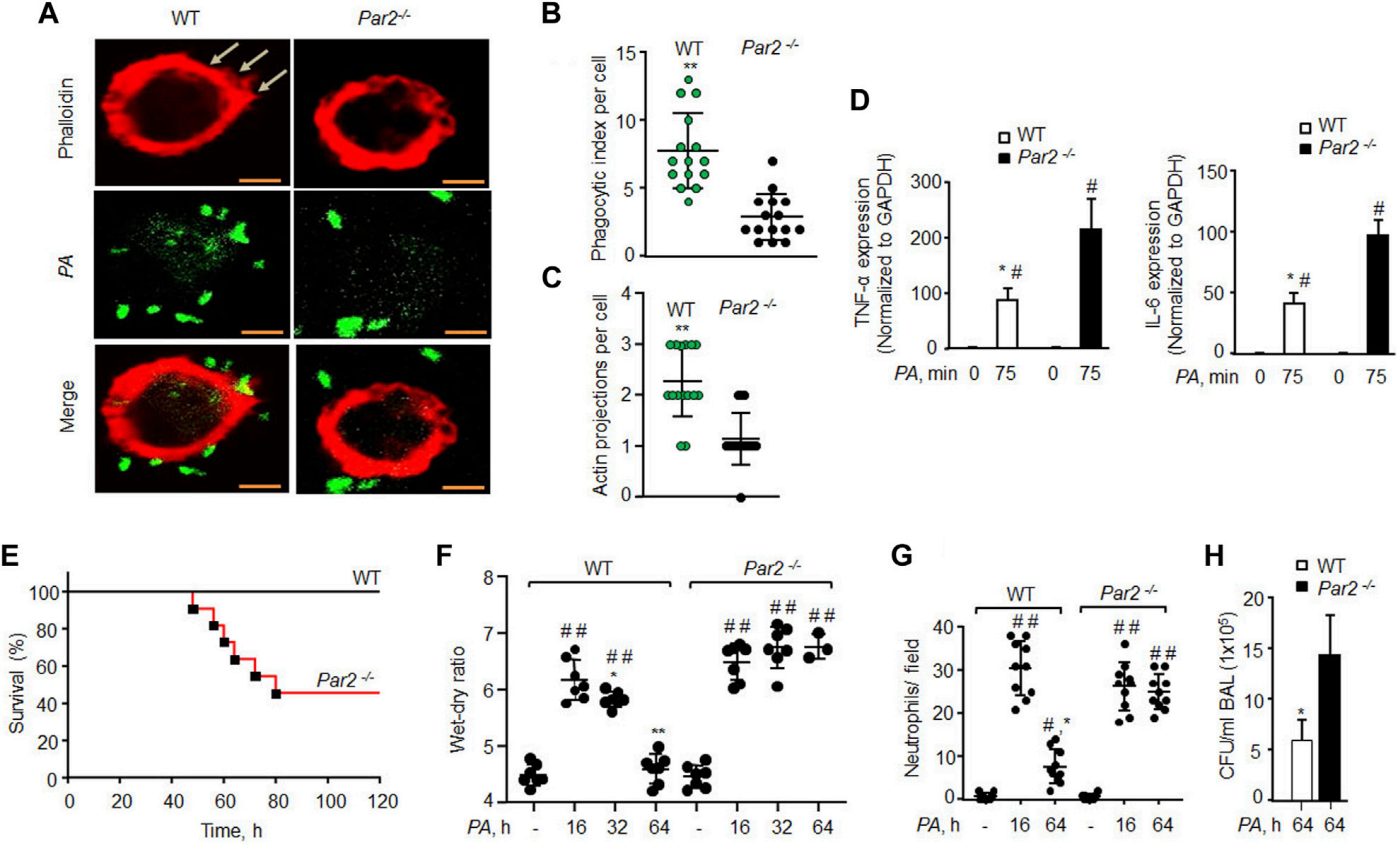
Loss of PAR2 compromises mouse recovery from bacterial pneumonia. **(A)** AMs isolated from BAL of mice exposed to *PA* (MOI,1:10) were fixed in 2% paraformaldehyde, stained with phalloidin, and imaged. Representative images are shown from three independently performed experiments. Bars, 5 micron meter. **(B)** Phagocytic index (of Figure 2A) was calculated by counting the total number of bacteria engulfed by a macrophage. Individual phagocytic index per macrophage is shown (n = 15 cells). **(C)** Protrusions (of Figure 2A) were quantified per macrophage. The data are shown as individual extensions per macrophage (n = 15 cells). **(D)** AMs were extracted from BAL and exposed to *PA* for 75 min followed by measurement of inflammatory cytokine gene expression by qPCR. The data are shown from three independent experiments. **(E)** Mouse survival was evaluated after administration of 1 × 10^6^ CFU of *PA* in WT and PAR2-null mice (n = 10 mice/group). **(F)** 1 × 10^6^ CFU of *PA* was injected into mice endotracheally followed by the assessment of the wet–dry ratio (n = 7 mice/group). **(G)** Neutrophil count (of Figure 2F) was performed from WT and PAR2-null mice post-*PA* infection. Individual values are shown from three independent experiments. **(H)** BAL was performed on *PA*-infected (1 × 10^6^ CFU) mice at 64 h, and CFUs of bacteria/ml of BAL fluid were counted. Data are shown as mean of three independent experiments. Data are presented as mean ± SD. *p < 0.05, **p < 0.001 relative to respective PAR2-null *PA*-exposed AMs or mice; #*p* < 0.05 relative to corresponding control of WT or PAR2-null AMs or mice; ##*p* < 0.001 relative to corresponding control of WT or PAR2-null mice. The analysis was performed using one-way ANOVA followed by a paired *t*-test.

AMs are phenotypically and genotypically different from BMDMs (Rayees et al., 2020). We thus isolated AMs from WT and PAR2-null lungs and exposed them to *PA*. We again found that PAR2-null AMs were defective protrusions in forming and *PA* phagocytosis (Figures 2A–C). Interestingly, *PA* markedly increased the expression of TNF-α and IL-6 in PAR2-null lung AMs relative to WT-AMs (Figure 2D). The same was true for WT and PAR2-null BMDMs exposed to *PA* (Supplementary Figure S2B). We also determined IL-1β and MIP-2 expression following *PA* infection and found a similar increase in their expression in both WT and PAR2-null BMDMs (Supplementary Figure S2B).

Bacterial pneumonia compromises patient survival in acute lung injury (Dreyfuss and Ricard, 2005; Rubenfeld et al., 2005). We instilled *PA* (1 × 10^6^ CFU/mouse) into WT and PAR2-null mice, and found that 60% of PAR2-null mice died within 64 h, while all WT mice survived **(** Figure 2E **)**. Upon sensing pathogens, AMs induce lung barrier leak and trigger neutrophil influx (Nagre et al., 2019; Rayees et al., 2019). We found that *PA* (1 × 10^6^ CFU/mouse) induced barrier dysfunction and neutrophil accumulation in the lungs at 16 h in both WT and PAR2-null mice with the same intensity. The WT mice resolved edema formation post 32 h *PA* infection. However, the surviving PAR2-null mice showed massive edema and increased neutrophil influx (Figures 2F,G). We also isolated BAL from the WT and PAR2-null mice at 64 h and quantified CFUs. PAR2-null lungs failed to clear bacteria after pneumonia since BAL isolated from PAR2-null lungs showed a ∼3-fold increase in CFU relative to WT lung BAL (Figure 2H, Supplementary Figure S2A).

### PAR2 mediates *PA* phagocytosis by activating Rac1GTPase

**FIGURE 3:**
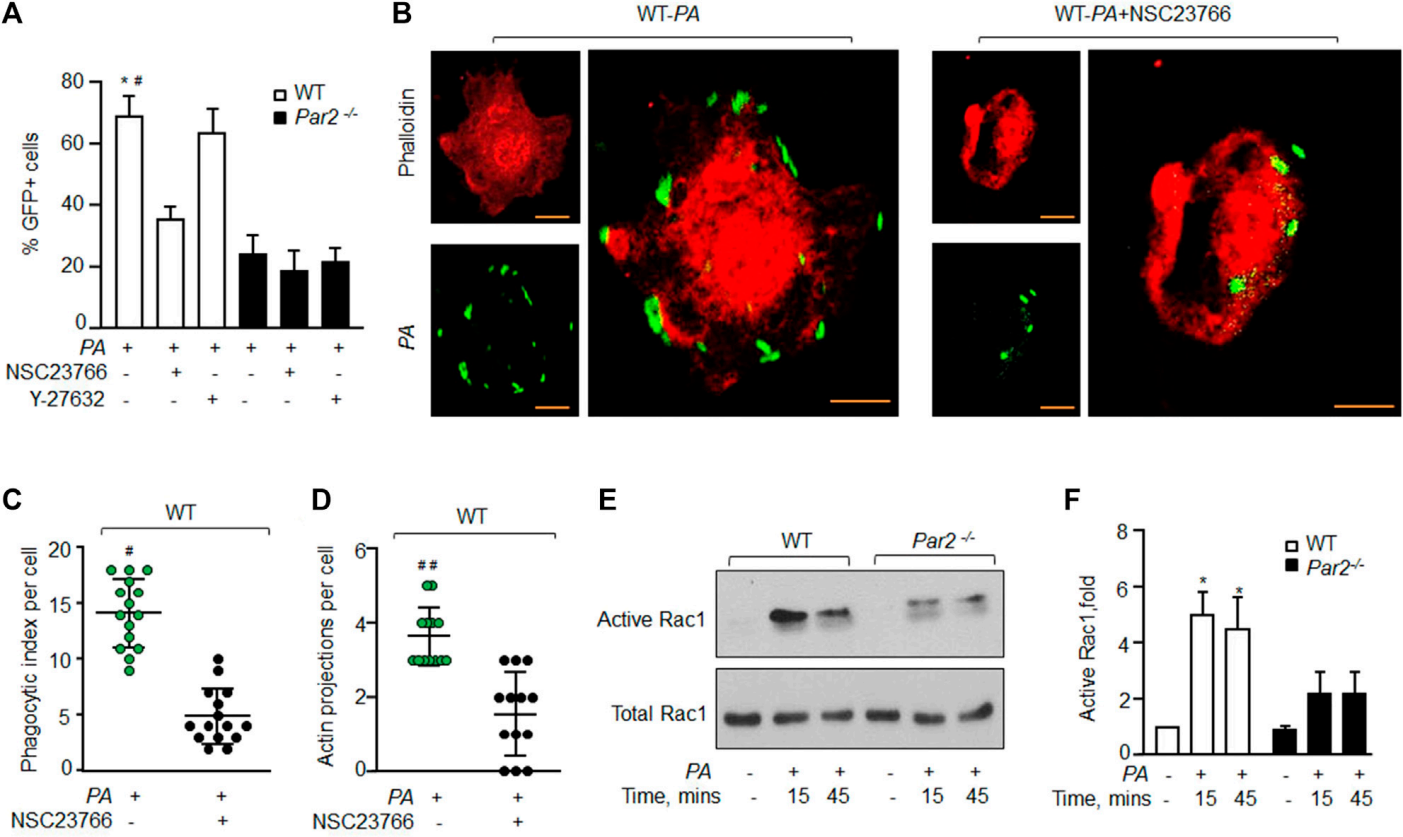
PAR2 mediates *PA* phagocytosis by activating Rac1. **(A)** WT and PAR2-null macrophages were pre-treated with 100 μM NSC23766 (Rac1 inhibitor) or 30 μM of Y-27632 (inhibitor of Rho-associated kinase) for 30 min after which they were exposed to *PA* for 45 min. The GFP^+^ cells were quantified by flow cytometric analysis. Data are shown as mean of three independent experiments. **(B)** WT-macrophages were pretreated with 100 μM NSC23766 for 30 min followed by *PA* exposure. The cells were fixed, stained with rhodamine-phalloidin, and imaged for the assessment of phagocytosis. Images shown are representative of three independently performed experiments. Bars, 5 micron meter, inset, zoomed ∼x2. **(C)** Phagocytic index (of Figure 3B) was calculated by counting the total number of bacteria engulfed by a macrophage. Individual count/phagocytic index per macrophage is shown (n = 15 cells). **(D)** Actin protrusions (of Figure 3B) were quantified per macrophage after the cells were fixed. The data are shown as individual extensions per macrophage (n = 13 cells). **(E)** WT and PAR2-null macrophages were exposed to *PA* for indicated times, and Rac1 activity was determined using GST-PAK beads. Total Rac1 was used as a loading control. A representative blot is shown from three independently performed experiments. **(F)** Densitometry plot (of Figure 3E) is shown. The data are shown as mean of three independently performed experiments (n = 3). In all figures, data are presented as mean ± SD. *p < 0.05 relative to PAR2-null *PA*-exposed macrophages; #*p* < 0.05, ##*p* < 0.001 relative to WT-macrophages treated with NSC23766. The analysis was performed using one-way ANOVA followed by a paired *t*-test.

Small GTPases of the Rho family, such as Rho A and Rac1, are known to regulate actin organization as well as the phagocytosis of several bacterial species, including *PA* (Lee et al., 2000; Kazmierczak and Engel, 2002; Flannagan et al., 2012; Levin et al., 2016). We, therefore, determined if PAR2-induced *PA* phagocytosis occurs by activating RhoA or Rac1. We pharmacologically inhibited RhoA and Rac1 signaling using specific small molecule inhibitors, after which *PA* phagocytosis was determined (Routhier et al., 2010; Levay et al., 2013). We found that the inhibition of Rac1 activity using NSC23766 (Levay et al., 2013) reduced the phagocytic index to ∼50% (Figure 3A and Supplementary Figure S3A). By contrast, the inhibition of RhoA signaling using Y-27632 (Routhier et al., 2010) had no effect on *PA* phagocytosis in WT or PAR2-null Mɸ (Figure 3A, Supplementary Figure S3A). These findings indicate that PAR2 ligation mediates the activation of Rac1, which then induces actin polymerization to promote phagocytosis.

To further corroborate these findings, we inhibited Rac1 activity in WT-Mɸ, which also prevented pseudopod formation and *PA* phagocytosis (Figures 3B–D and Supplementary Figure S3B-C). We next measured the Rac1 activity in WT and PAR2-null BMDMs following *PA* exposure. We observed that Rac1 activity was barely detectable in naïve WT-Mɸ. *PA* exposure increased Rac1 activity by ∼5-fold within 15 min, consistent with robust *PA* phagocytosis seen in Figure 1. Rac1 activity declined at 45 min (Figures 3E,F). Interestingly, *PA* only modestly increased Rac1 activity in PAR2-null Mɸ. Thus, Rac1 activity was significantly lower in PAR2-null Mɸ at 15 and 45 min than in WT-Mɸ.

We also addressed the possibility that loss of PAR2 in AMs induced their apoptosis following infection. Thus, we determined the expression of caspase 11 and PARP1 (two well-known markers of apoptosis), (Chaitanya et al., 2010; Huang et al., 2019). In WT and PAR2-null macrophages following *PA* infection. We failed to detect any observable differences in the expression of these proteins between the two groups (Supplementary Figure S3D). We also performed annexin V staining, another marker of cell death, (Bossy-Wetzel and Green, 2000) in WT or PAR2-null macrophages after *PA* infection. We again did not observe any significant apoptosis of macrophages at the indicated time (Supplementary Figure S3E).

### PAR2 mediates Rac1 activity by enhancing cAMP levels

**FIGURE 4:**
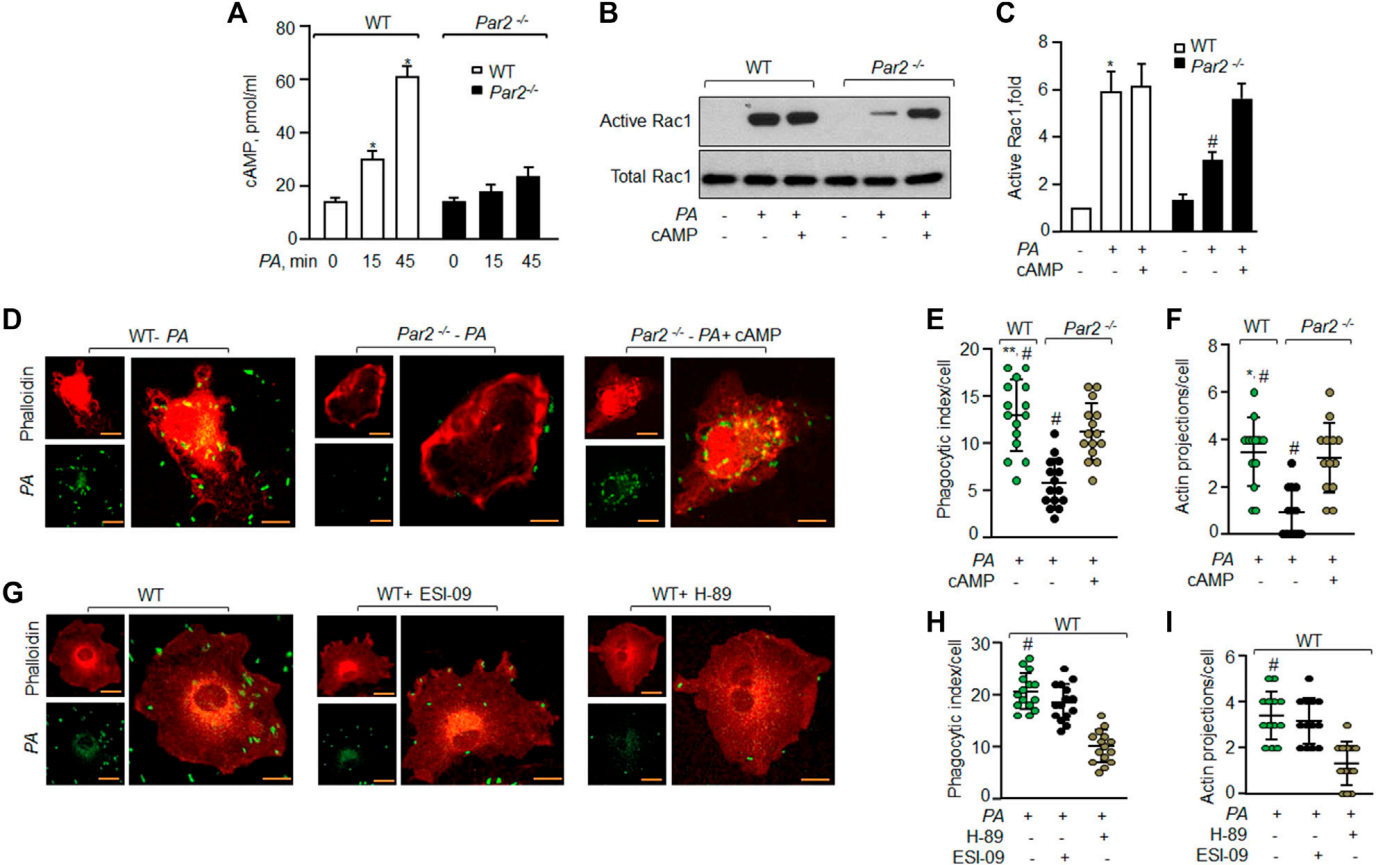
PAR2 induces cAMP that activates Rac1 to phagocytize *PA*. **(A)** WT and PAR2-null macrophages were exposed to *PA* for indicated times. The cells were lysed and intracellular cAMP was measured using ELISA (n = 3). **(B)** WT and PAR2-null macrophages were pre-treated with 10 nM of 8-Br-cAMP for 30 min followed by exposure to *PA* for 45 min and Rac1 activity was determined. Total Rac1 was used as the loading control. A representative blot is shown from three independently performed experiments. **(C)** Densitometry plot (of Figure 4B) is shown. The data are presented as mean of three independently performed experiments. **(D)** PAR2-null macrophages were pre-treated with 10 nM of 8-Br-cAMP for 30 min followed by exposure to *PA* for 45 min. The cells were fixed and stained with rhodamine-phalloidin. Images were acquired to quantify phagocytosis. The images shown are representative of results from three independently performed experiments. Bars, 5 micron meter, inset, zoomed ∼x2. **(E)** Phagocytic index (of Figure 4D) was calculated by counting the total number of bacteria engulfed by a macrophage. Individual count/phagocytic index per macrophage is shown (n = 15 cells). **(F)** Protrusions were quantified per macrophage (of Figure 4D) as described previously. Individual extensions per macrophage are shown (n = 13 cells). **(G)** WT macrophages were pre-treated with 20 μM of ESI-09 or 10 μM of H-89 for 30 min followed by exposure to *PA* for 45 min. The cells were fixed, stained with rhodamine-phalloidin and imaged for the assessment of phagocytosis. Representative images are shown from three independently performed experiments. Bars, 5 micron meter, inset, zoomed ∼x2. **(H)** Phagocytic index (of Figure 4G) was calculated by counting the total number of bacteria engulfed by a macrophage. The individual phagocytic index per macrophage is shown (n = 15 cells). **(I)** Protrusions (of Figure 4G) were quantified per macrophage. Individual extensions per macrophage are shown (n = 13 cells). Data are presented as mean ± SD. *p < 0.05, **p < 0.001 relative to respective PAR2-null *PA*-exposed BMDM; #*p* < 0.05 relative to WT BMDM or PAR2-null BMDM treated with H-89 or 8-Br-cAMP, respectively. The analysis was performed using one-way ANOVA followed by a paired *t*-test.

We have recently shown that the ligation of PAR2 with thrombin in macrophages induces cAMP generation (Rayees et al., 2019). cAMP is known to regulate Rac1GTPase activity (Schlegel and Waschke, 2013; Aslam et al., 2014). Thus, we tested the possibility that PAR2 stimulates cAMP generation, which activates Rac1 to mediate *PA* phagocytosis. Interestingly, *PA* increased cAMP levels by 2-fold within 15 min and 4-fold at 45 min in WT-Mɸ. *PA* failed to increase cAMP levels in PAR2-null Mɸ above baseline (Figure 4A).

Next, we pre-treated PAR2-null Mɸ with cell-permeable cAMP (8-Br-cAMP) to test the prediction that augmenting cAMP levels in PAR2-null Mɸ should rescue Rac1 activity and phagocytosis. We found that treatment of PAR2-null Mɸ with the cAMP analog restored the Rac1 activity (Figures 4B,C) and phagocytosis (Figures 4D–F) to the level seen in WT Mɸ. These findings demonstrate that *PA* activates PAR2, which induces cAMP generation to activate Rac1.

cAMP activates PKA and EPAC to signal Rac1 activation (Cheng et al., 2008; Birukova et al., 2010; Bachmann et al., 2013). Thus, we inhibited PKA and/or EPAC activity in WT-Mɸ and found that inhibition of PKA but not EPAC reduced phagocytosis significantly (Figures 4G–I).

### Delivery of constitutively active Rac1 in alveolar macrophages resolves lung infection and reduces mortality

**FIGURE 5:**
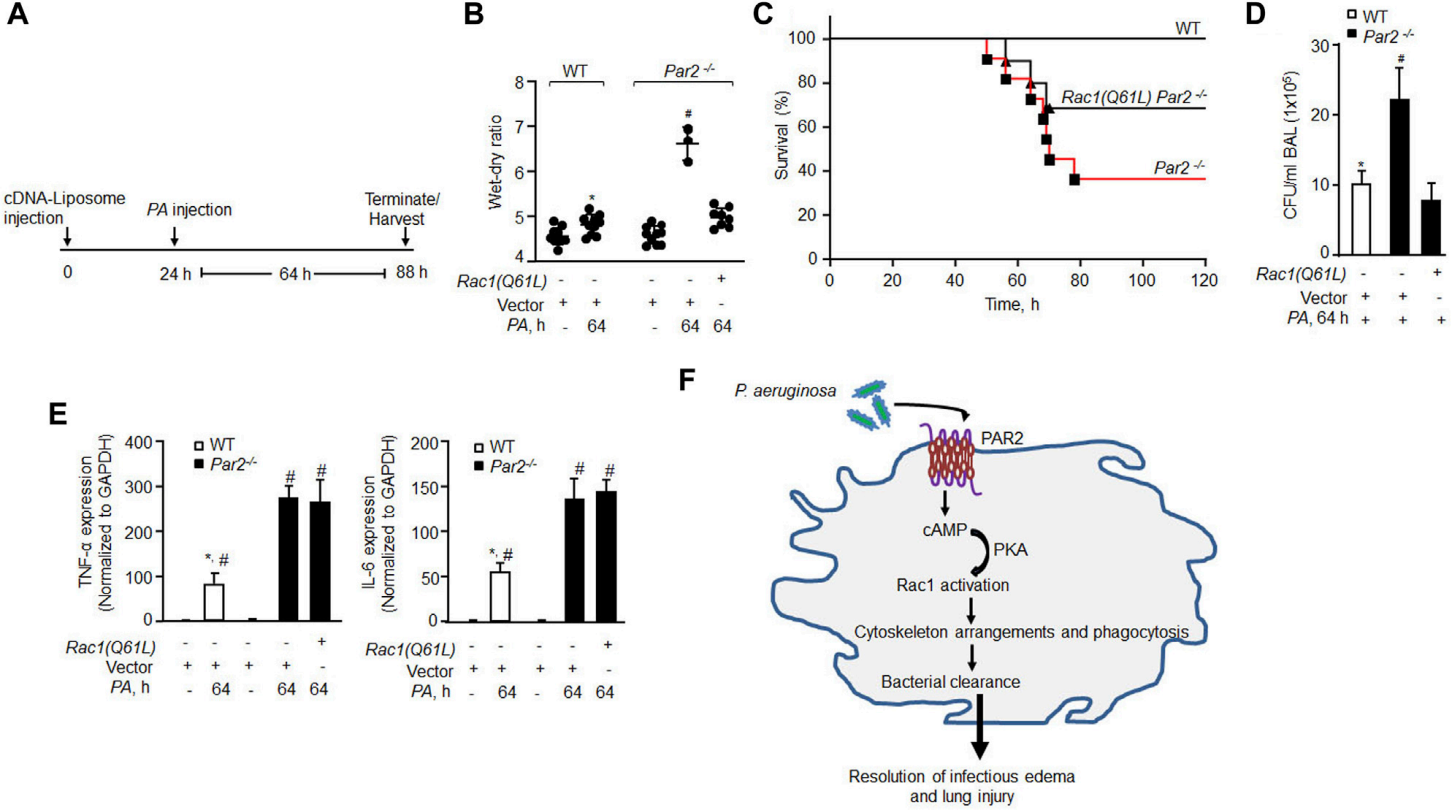
Delivery of constitutively active Rac1 rescued bacterial clearance leading to resolution of lung injury and mouse survival from bacterial pneumonia. **(A)** Schematics of the protocol. Liposomes complexed with constitutively active Rac1 (CA-Rac1; Q61L) or vector were administrated endotracheally and after 24 h *PA* was injected, and lung injury and mice survival were determined at indicated times. **(B)** Lung wet–dry ratio was determined to assess lung edema (n = 10 mice/group; only three mice are shown for vector Pa*r2* ^*−/−*^ group due to 70% mortality in this group). **(C)** Mouse survival after *PA* (1 × 10^6^ CFU) administration (n = 10 mice/group). **(D)** BAL was isolated from mice (from Figure 5B) at 64 h post infection, and CFU of bacteria/ml of BAL fluid was counted. Data are shown from three independent experiments. **(E)** AMs were isolated from BAL of the indicated mice, and the expression of TNF-α and IL-6 was measured from the indicated groups by qPCR (n = 3). **(F)** Model of PAR2-mediated *PA* phagocytosis in AMs and resolution of acute lung injury and the resultant lethality. *PA* activates PAR2 in AMs stimulating cAMP generation. cAMP stimulates PKA activity which in turn activates Rac1. Activated Rac1 induces actin cytoskeleton remodeling leading to phagocytosis of *PA* resolving lung injury after bacterial pneumonia. Data are presented as mean ± SD. *p < 0.05 relative to respective *PA*-infected *Par2* ^*−/−*^ mice or AMs; #*p* < 0.05 relative to respective *Par2* ^*−/−*^ Rac1(Q61L) + *PA*-infected mice or control PAR2-null AMs. The analysis was performed using one-way ANOVA followed by a paired *t*-test.

Based on our observation that constitutively active Rac1 restored *PA* phagocytosis in AMs lacking PAR2 expression*,* we surmised that rescuing constitutive Rac1 would clear infection and thereby mitigate lung injury. Indeed, we observed that constitutively active Rac1 (CA-Rac1) mutant restored Rac1 activity in PAR2-null BMDMs (Supplementary Figure S4A, B). Thus, we delivered vector or YFP-tagged CA-Rac1 in mouse lungs, using liposomes. After 48 h, we injected unlabeled *PA* and determined the protrusive activity of Rac1 in AMs 3 h later using Rac1 and actin antibodies. We found that CA-Rac1-receiving AMs formed numerous pseudopods upon *PA* infection than WT-AMs (Supplementary Figure S4D, E). Importantly, CA-Rac1-receiving PAR2-null mice resolved lung edema (Figures 5A,B). Moreover, CA-Rac1 delayed the mortality of PAR2-null mice from pneumonia such that 70% of PAR2-null mice survived at 120 h post bacterial challenge than PAR2-null mice receiving control vector (Figure 5C). Survived PAR2-null mice receiving CA-Rac1 also showed decreased CFU counts (Figure 5D, S4C). However, the restoration of Rac1 activity failed to reduce the expression of pro-inflammatory cytokines to the levels observed in WT-AMs post *PA* challenge (Figure 5E), indicating that the augmented expression of TNF-α and IL-6 in *PA* exposed PAR2-null AM (Figure 5E) is independent of Rac1 activation and hence not related to phagocytosis in the present study model.

## Discussion

Bacterial pneumonia caused by *PA* remains a concern for critically ill patients suffering from conditions such as cystic fibrosis and critically ill ALI patients in the ICU despite new-generation antibiotics (Sadikot et al., 2005; Malhotra et al., 2019). AMs are the first line of defense against lung bacterial and viral infections (Hashimoto et al., 2007; Kolli et al., 2014; Dinapoli et al., 2017; He et al., 2017). How then AMs become inefficient in clearing *PA*? Alteration in PAR2 expression occurs in several inflammatory diseases (Yasuoka et al., 1997; Knight et al., 2001; Miotto et al., 2002). We showed that the loss of PAR2 expression in AMs reduced *PA* phagocytosis by half, leading to impaired bacterial clearance and irreversible lung injury, consistent with the previous study (Moraes et al., 2008). Thus, 60% of the PAR2-null mice died after lethal pneumonia. Interestingly, PAR2-null AMs generated significantly higher levels of inflammatory cytokines than the WT AMs and triggered neutrophil influx. Neutrophils recruited to the airspace are ineffective in clearing *PA* (Mishra et al., 2012; Thanabalasuriar et al., 2017). We showed that PAR2 expressed in AMs contributed to *PA* clearance and lung homeostasis. Our studies, however, did not rule out the role of PAR2 in neutrophils in co-operating with AM-PAR2 in regulating *PA* phagocytosis.

AMs trigger phagocytosis by mobilizing the scavenger receptors such as MARCO and Fc receptors (FcγRs) (Bonilla et al., 2013; Levin et al., 2016). We showed that about ∼60% of *PA* phagocytosis was PAR2-dependent. These findings also showed that the supernatant from *PA*-infected PAR2-null AMs formed more colonies, pointing to defective bacterial internalization. Whether AM-PAR2 regulated phagocytosis by maintaining MARCO and Fc receptor functions remains to be seen.

Phagosome formation aided by rapid actin remodeling is a crucial step in internalizing bacteria (Levin et al., 2016). RhoA and Rac1 regulate actin remodeling (Sit and Manser, 2011; Flannagan et al., 2012). In line with these studies, we showed that inhibiting actin polymerization or Rac1 activity blocked *PA* phagocytosis in WT macrophages. However, RhoA was not required. Intriguingly, *PA* activated Rac1 in WT-AMs, but not in PAR2-null AMs. Hence, AMs lacking PAR2 showed impaired pseudopod formation and defective phagocytosis, indicating that PAR2 induced phagocytosis by activating Rac1-mediated actin polymerization. Furthermore, delivery of constitutively active Rac1 rescued phagocytosis in PAR2-null lungs *in vivo* leading to 70% survival of the PAR2-null mice after *PA* challenge. The data presented in this study demonstrated an essential role for Rac1 but not RhoA downstream of PAR2 in phagocytizing *PA*. The requirement for active Rac1 in bacterial phagocytosis is consistent with its role in actin assembly for the ingestion of Gram-negative pathogens (Lee et al., 2000; Skjesol et al., 2019).

How does PAR2 induce Rac1 activity in macrophages upon *PA* infection? During tissue injury, PAR2 activation occurs by proteases such as trypsin, thrombin, and elastin due to the generation of tethered ligands (Zhao et al., 2015; Rayees et al., 2019; Rayees et al., 2020). However, elastase released from *PA* prevented PAR2 from being activated by trypsin (Dulon et al., 2003). Additionally, *PA* may trigger phagocytosis by generating matrix metalloproteinases, such as MMP-1, MMP-8, and MMP-13, which also cleave PAR2 (Falconer et al., 2019). Further studies are needed to explore *via* which of these proteases *PA* activated PAR2.

Controversy exists regarding the role of cAMP in regulating phagocytosis. Makranz et al. (2006) showed that cAMP promoted phagocytosis of myelin by glial macrophages. In sharp contrast, Serezani et al. (2007) showed that cAMP generation downstream of PGE2-coupled EP2/EP4 receptors suppressed the microbicidal activity of *Klebsiella pneumoniae* by AMs. However, mice lacking EP2 receptors showed enhanced phagocytosis of *PA* (Sadikot et al., 2005). In the present study, we also showed a critical role for cAMP as the second messenger in regulating the Rac1 activity and actin cytoskeletal reorganization, which induces *PA* phagocytosis. We showed that *PA* infection induced cAMP generation in macrophages in a PAR2-dependent manner. Specifically, we showed that cAMP addition to PAR2-null macrophages rescued the Rac1 activity and *PA* phagocytosis. cAMP activates PKA and EPAC which in turn induce Rac1 activity (Cheng et al., 2008; Birukova et al., 2010). We showed that the inhibition of PKA reduced the phagocytosis of *PA* in WT macrophages, indicating that PAR2 activated Rac1 via PKA.

Precisely how Rac1 was activated by PKA downstream of PAR2 to mediate phagocytosis is unknown, although it likely involves either the inhibition of guanine dissociation inhibitor (GDI) or the activation of guanine nucleotide exchange factors (GEFs) (Chahdi and Sorokin, 2008; Qiao et al., 2008). A likely scenario is the inhibition of GDI because PKA limits GDI inhibitory activity on Rac1 (Qiao et al., 2008). Another possibility is that PKA augments Rac1 activity through Vav1, a characterized GEF for Rac1 and phagocytosis (Patel et al., 2002).

Phagocytosis *per se* regulates pro-inflammatory gene expression (Freeman and Grinstein, 2016; Kaufmann and Dorhoi, 2016). In the present study, we established the role of PAR2 in regulating phagocytosis in association with an increase in Rac1 activity, which proved essential for the phagocytic capacity of macrophages. Thus, we showed that PAR2-null AMs have low Rac1 activity and produce cytokines at much higher levels than WT-AMs presumably because bacterial clearance becomes ineffective in these knockout mice. Accordingly, delivery of constitutively active Rac1 to PAR2-null mice rescued phagocytosis. Our findings do not rule out the involvement of other cell types, such as neutrophils, in this response. However, AMs are the most sentinel cells in the lungs and mediate phagocytosis of bacteria and viruses (Hashimoto et al., 2007; Dinapoli et al., 2017). Compared to other lung cell types, AMs predominantly expressed Rac1 (Nicholas et al., 2018). We showed that RhoA had no direct role in regulating *PA* phagocytosis but may mediate pro-inflammatory cytokine expression in macrophages when bacterial clearance is low (Manukyan et al., 2009; Yang et al., 2014). We concluded that the function of PAR2 in AMs is to activate Rac1, thereby facilitating *PA* phagocytosis, whereas RhoA may augment cytokine generation, leading to inflammatory injury (Popoff, 2014; Abedi et al., 2020). Thus, our findings show the importance of Rac1 activity in AMs in *PA* phagocytosis and thereby in resolving lung injury.

In summary, we demonstrated an essential role of the AM-PAR2-mediated cAMP-Rac1 pathway in triggering phagocytosis of *PA* (Figure 5F). We showed that loss of PAR2 expression and consequent impairment of Rac1 activity compromised *PA* clearance in the PAR2-null lungs, resulting in ALI and death from bacterial pneumonia. Liposomal delivery of constitutively active Rac1 in PAR2-null mice rescued phagocytosis and eliminated the lethality of *PA.* PAR2 in macrophages may have future therapeutic significance as a strategy to promote *PA* phagocytosis to treat bacterial pneumonia.

## Data Availability

The original contributions presented in the study are included in the article/supplementary material; further inquiries can be directed to the corresponding author.
