# Supplementary material for: Protease-activated receptor 2 promotes clearance of Pseudomonas aeruginosa infection by inducing cAMP-Rac1 signaling in alveolar macrophages
Source: Front Pharmacol. 2022 Sep 20;13:874197. doi: 10.3389/fphar.2022.874197 (PMC9530345; doi:10.3389/fphar.2022.874197)
Supplement: Supplementary file 1 [file DataSheet1.pdf]

Supplemental Information

Legends to supplementary figures

Figure S1: (Related to Figure 1)

(A) WT or PAR2 null macrophages were pretreated with Latrunculin A (20nM) for 30 min followed by exposure to *PA* for 45 mins. The macrophages were treated with accutase, spun down, and washed. GFP^{+} cells were quantified using flowcytometry. A representative contour plot is shown from three independently performed experiments.

Data are presented as mean ± SD. The analysis was performed using one-way ANOVA followed by paired t-test.

Figure S2: (Related to Figure 1 and 2)

(A) BAL was spread on blood agar plates and after 24 h *PA* colonies (CFU) were counted. Representative photographs of blood agar plates are shown from three independent experiments.
(B) WT and PAR2 null BMDM were exposed to *PA* for 75 minutes followed by measurement of inflammatory cytokine gene expression by qPCR (n=3).

Data are presented as mean ± SD. *$p < 0.05$ relative to *PA* exposed PAR2 null BMDM, #$p < 0.05$ relative to respective untreated (control) cells. The analysis was performed using paired t-test.

Figure S3: (Related to Figure 3)

(A) WT and PAR2-null macrophages were pretreated with NSC23768 (100µM) or Y727832 (30µM) for 30 mins followed by exposure to GFP^{+} *PA* for 45 mins. Representative contour plot of three independent experiments is shown.
(B) WT BMDM were pretreated with 100µM Rac1 inhibitor for 30 mins followed by *PA* exposure and Rac1 activity was determined using GST-PAK beads. Total Rac1 was used to as a loading control. A representative blot of three independent experiments is shown.
(C) Densitometry plot (of figure S3B) is shown. The data are presented as mean of three independent experiments.
(D) Immunoblot showing Caspase 11 and cleaved PARP expression following *PA* exposure. The experiment was performed three times. Representative blots are shown in the figure.
(E) Annexin V positive cells were quantified by flow cytometry following *PA* exposure. Representative contour plot of three independent experiments is shown.
(F) The percentage of Annexin V positive macrophages following *PA* exposure is shown. The data represents mean± SD of three experiments (of Fig S3E).

Data are presented as mean ± SD. *$p < 0.05$ relative to respective *PA* exposed WT BMDM treated with NSC23766, #$p < 0.05$ relative to untreated WT BMDM. The analysis was performed using one-way ANOVA followed by paired t-test.

Figure S4: (Related to Figure 5)

(A) WT and PAR2 null macrophages were transfected with vector or CA-Rac1(Q61L) cDNA and 48 h later, the cells were exposed to *PA* for 45 min. Total and active Rac1 were assessed by western blotting. Representative blot of three independently performed experiments is shown.
(B) Densitometry plot (of figure S4A) is shown. The data are presented as mean of three independent experiments.
(C) BAL from the indicated group of mice was performed at 64 h post-infection and CFU of bacteria/ml of BAL fluid were counted. Representative photographs of blood agar plates used for *PA* CFU count (n=3) are shown.
(D) *PA* ($1x10^6$ CFU) were administered 48 h after liposome administration intratracheally and AMs were extracted 3h later. Cells were fixed, stained with Siglec-F, actin and Rac1 antibodies. Images were acquired using confocal microscope. Bars, 5-micron meter, inset, zoomed ~x3. The images are representative of results from three independently performed experiments.
(E) Pseudopods were quantified by measuring spatial distribution of Rac1/ F-actin fluorescent intensity peaks near cell edges (n=12 cells/group). Experiments were performed at least two times independently.

Data are presented as mean ± SD. *p < 0.05 relative to respective *PA* exposed PAR2 null BMDM and/or AM, #p < 0.05 relative to respective untreated/control WT or PAR2 null BMDM. The analysis was performed using one-way ANOVA followed by paired t-test.

Supplemental Methods

Apoptosis of BMDM following *PA* exposure

The apoptosis was assessed in WT and PAR2 null BMDM following *PA* (MOI, 1: 10) exposure for 45 minutes. The macrophages were then washed with PBS and lifted off the plate by accutase treatment. This was followed by a wash with PBS and another wash with Annexin binding buffer. The cells were then stained with 5µL of Annexin V-EF450 in 100µL of Annexin binding buffer per sample for 15 minutes at room temperature. Cells were then washed with the Annexin binding buffer and resuspended in 200µL of the same buffer and 5µL of 7-AAD was added. Samples were recorded within 15 minutes by flow cytometer after 7-AAD addition.

We also assessed the apoptosis by measuring caspase-11 and cleaved PARP by western blotting. BMDM were exposed to *PA* at MOI, 1: 10 for 15 and 45 minutes. The cells were washed with PBS, lysed using RIPA lysis buffer containing 1% protease inhibitor cocktail. The lysates were dissolved in Laemmli buffer and caspase 11 and PARP were assessed by immunoblotting.

Figure S1

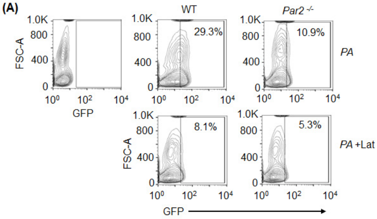

(A)
WT
Par2 -/-
1.0K
800
400
0
FSC-A
29.3%
10.9%
PA
8.1%
5.3%
PA +Lat
10⁰
10²
10⁴
GFP

Figure S2

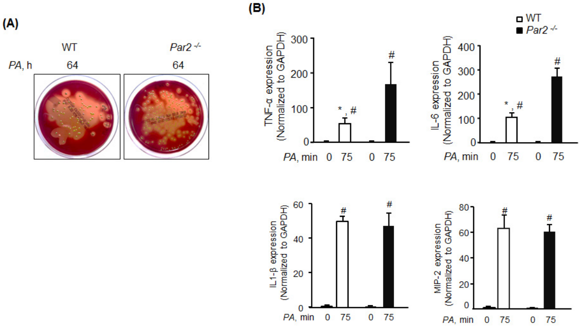
(A)
WT
Par2 -/-
PA, h
64
64
(B)
WT
Par2 -/-
TNF-α expression (Normalized to GAPDH)
IL-6 expression (Normalized to GAPDH)
IL1-β expression (Normalized to GAPDH)
MIP-2 expression (Normalized to GAPDH)
PA, min
0
75
*, #
#

Figure S3

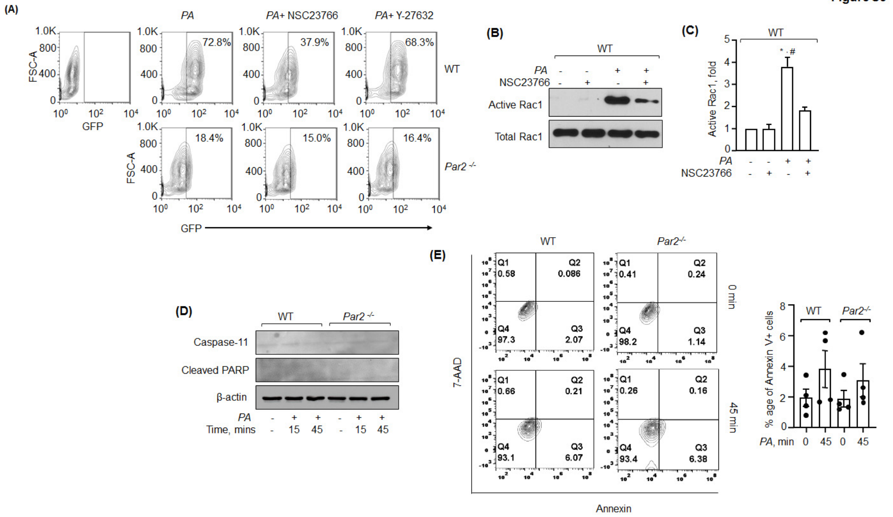
(A)
PA
PA+ NSC23766
PA+ Y-27632
72.8%
37.9%
68.3%
WT
18.4%
15.0%
16.4%
Par2 -/-
FSC-A
GFP
(B)
WT
PA
NSC23766
Active Rac1
Total Rac1
(C)
WT
Active Rac1, fold
PA
NSC23766
(D)
WT
Par2 -/-
Caspase-11
Cleaved PARP
β-actin
PA
Time, mins
15
45
(E)
WT
Par2-/-
Q1 0.58
Q2 0.086
Q4 97.3
Q3 2.07
Q1 0.41
Q2 0.24
Q4 98.2
Q3 1.14
0 min
Q1 0.66
Q2 0.21
Q4 93.1
Q3 6.07
Q1 0.26
Q2 0.16
Q4 93.4
Q3 6.38
45 min
7-AAD
Annexin
% age of Annexin V+ cells
PA, min

Figure S4

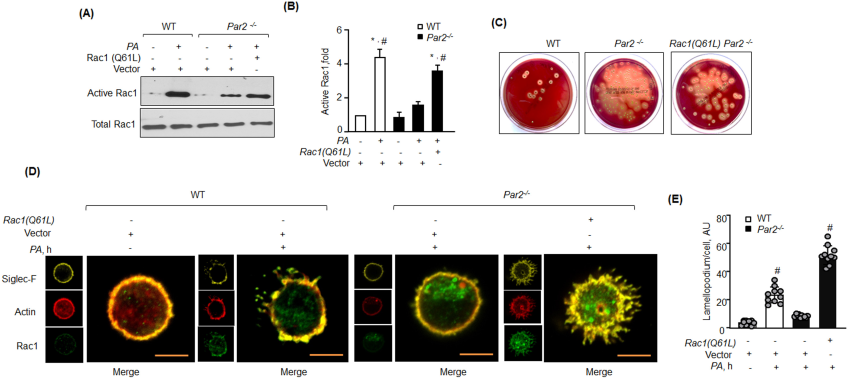

(A)
WT
Par2 -/-
PA
Rac1 (Q61L)
Vector
Active Rac1
Total Rac1
(B)
Active Rac1,fold
WT
Par2-/-
*,#
PA
Rac1(Q61L)
Vector
(C)
WT
Par2 -/-
Rac1(Q61L) Par2 -/-
(D)
WT
Par2-/-
Rac1(Q61L)
Vector
PA, h
Siglec-F
Actin
Rac1
Merge
(E)
Lamellopodium/cell, AU
WT
Par2-/-
#
Rac1(Q61L)
Vector
PA, h
